# Analysis of Fluency of Movement in Parkour Using a Video and Inertial Measurement Unit Technology

**DOI:** 10.5114/jhk/166581

**Published:** 2023-06-05

**Authors:** Francesco Feletti, Cristian Bracco, Takeko Maria Molisso, Lorenzo Bova, Andrea Aliverti

**Affiliations:** 1 Department of Electronics, Information and Bioengineering, Politecnico di Milano, Milan, Italy.; 2 Department of Radiology, Ausl Romagna, S. Maria delle Croci Hospital, Ravenna, Italy.; 3 Department of Translational Medicine and for Romagna, Università degli Studi di Ferrara, Ferrara, Italy.; 4 Department of Industrial Engineering (DII), University of Padua, Padova, Italy.; 5 UCLA Department of Orthopaedic Surgery, David Geffen School of Medicine, Los Angeles, California, USA.

**Keywords:** motor skills, injury, training, athletic gestures, sports science

## Abstract

Fluency is a movement parameter combining smoothness and hesitation, and its objective measurement may be used to determine the effects of practice on sports performance. This study aimed to measure fluency in parkour, an acrobatic discipline comprising complex non-cyclical movements, which involves fluency as a critical aspect of performance. Inter-individual fluidity differences between advanced and novice athletes as well as intra-individual variations of fluency between different parts and subsequent repetitions of a path were addressed. Seventeen parkour participants were enrolled and divided into two groups based on their experience. We analysed signals captured from an inertial measurement unit fixed on the back of the pelvis of each participant during three consecutive repetitions of a specifically designed parkour routine under the guidance of video analysis. Two fluency parameters, namely smoothness and hesitation, were measured. Smoothness was calculated as the number of inflexions on the so-called jerk graph; hesitation was the percentage of the drop in the centre of mass velocity. Smoothness resulted in significantly lower values in advanced athletes (mean: 126.4; range: 36–192) than in beginners (mean: 179.37; range: 98–272) during one of the three motor activities (p = 0.02). A qualitative analysis of hesitation showed that beginner athletes tended to experience more prominent velocity drops and negative deflection than more advanced athletes. In conclusion, a system based on a video and an inertial measurement unit is a promising approach for quantification and the assessment of variability of fluency, and it is potentially beneficial to guide and evaluate the training process.

## Introduction

The fluency of movement is a parameter used mainly in the clinical setting. It is used to evaluate neurological rehabilitation because it combines movement characteristics that escape more traditional kinematic measurements (Dion et al., 2013; Kerr et al., 2013). The fluency of a movement is also a fundamental characteristic of performance in the sport of parkour. In both contexts, the determination of fluency is limited by subjectivity being generally judged visually, applying a standard scale (Pomeroy et al., 2003) or scoring cards (Dvořák et al., 2018).

Some attempts have been made to obtain a more objective measure of the fluency of movement, including the analysis of speed reduction (Dion et al., 2013). However, the speed reduction is only one feature of fluency, which is a complex parameter that also involves smoothness and hesitation (Kerr et al., 2013). While the speed reduction is probably sufficient to evaluate elementary gestures in the neuro-rehabilitation setting, it is a simplistic analysis that cannot be adequate in sport. Indeed, in sports, a *movement* is defined as fluid when it propagates effectively along the kinematic chains, minimizing energy dissipation (Piana et al., 2016). Sport gestures involve coupling perception and action through mutual relationships along the kinematic chains.

Fluency was included among the determinants of sports technique in a recent overview of race-walking biomechanics (Pavei et al., 2014). Moreover, since fluency incorporates both spatial (i.e., trajectory) and temporal parameters, its measurement has been proposed by Seifert et al. (2014) as a tool to evaluate the effects of practice on sports performance. Specifically, those authors studied eight experienced climbers wearing a single magnetic and inertial measurement unit (MIMU) at a 100 Hz sample frequency located at the hip to compute a jerk (the third derivative of position regarding time) as a smoothness measure. Athletes participated in four testing sessions in that study, each consisting of two distinct route ascents of different complexity. However, fluency was explored through jerk analysis only, without considering other possible parameters such as hesitation. Other studies used accelerometers to create meaningful metrics of body acceleration from a MIMU (Minami et al., 2011), including some earlier sport climbing experiences (Pansiot et al., 2008).

Using fluency for performance and training evaluation can be a promising approach, especially in acrobatic disciplines involving complex non-cyclical movements, such as parkour. Parkour was defined as “the art of moving from one point to another respecting a key principle: efficiency and fluency” (International Gymnastics Federation, 2019), and since achieving the fluency of the movement represents the highest step of the experience in this sport, it is worth exploring the fluency of movement in parkour, and how this parameter evolves with experience and practice (Bartlett et al., 2006). On the one hand, since it is one of the fundamental principles mentioned in the definition of parkour, fluency might be an element of objective evaluation of this sport discipline. For example, the measurement of fluency was included among the determinants of a scale from 0 to 45 for an objective parkour technique developed by Dvorak et al. (2018). On the other hand, in contrast with more traditional sports, which pursue the achievement of quantitative goals (time, score, order of arrival), where fluidity may be unnecessary to achieve the goal, pursuing higher levels of movement fluency is a distinctive element of high-performance quality in parkour. This characteristic makes parkour more similar to gymnastics and acrobatics, where fluency is among the valuable features in evaluating movement (Król et al., 2020), and techniques adopted to perform athletic gestures specifically aim at improving fluency (Takei and Dunn, 1996). Indeed, in parkour, aesthetic criteria are at the core of performance evaluation, and the sport is further developing in this direction among younger communities of participants.

Although optical systems are the gold standard for evaluating lower limb function during the gait, they have limitations in motion analysis applied to sports: the lack of standardized protocols, limited acquisition volume, the need for calibration, synchronization and configuration, cost, portability, interference with bright sunlight whenever outdoors, as well as the necessity for a clear line of sight (Van der Kruk, 2018). Besides, some specific difficulties in approaching motion analysis in parkour derive from this sport's gestures' acrobatic and non-cyclical nature. Some setups based on MIMUs have proven to be a valid and economical alternative to optical systems in a wide range of sports applications: from injury prevention to on-field performance evaluation (Camomilla et al., 2018).

Recently, MIMUs have been applied to the measurement of smoothness as an indicator of fluency in the sport of climbing (Seifert et al., 2014). Although climbing lacks the sudden acceleration characteristics of parkour, both disciplines require complex and non-cyclical movements; moreover, climbing is one of the basic athletic gestures in parkour.

The present study explores the possibility of directly measuring the parkour's fluency based on video+MIMU technology. Specifically, since movement properties can change with experience (Seifert et al., 2013), the main objective of this study was to establish whether the obtained quantitative measure of fluency was accurate enough to highlight significant differences between advanced and novice athletes. The primary hypothesis was that a measuring system based on a single MIMU would allow to measure a higher level of smoothness and a lower level of hesitation among advanced athletes compared to beginners when performing a path made of three typical parkour movements.

Concurrently, since aggregated data are of limited value in studying movement patterns (Kelso, 2005), this study also addresses an intra-individual approach to fluency variability to highlight subtle movement individualities or 'signature' patterns functional to the execution of sports motor tasks (Davids et al., 2003). The secondary hypothesis was, therefore, that a measurement system based on a video and a MIMU could grasp differences in intra-individual variability of movement along subsequent repetitions of the parkour routine between advanced athletes and beginners.

## Methods

### Participants

We enrolled 17 male parkour participants (a.k.a. traceurs) from two classes of a parkour school: eight were recruited from beginners and nine from the more advanced. The number of participants was chosen by analogy with previous studies (Seifert et al., 2014). Students belonging to two different classes imply a skill level assessment had been carried out by the school staff, composed of Art du Déplacement And Parkour Teaching (ADAPT) certificated instructors (https://adaptqualifications.com). An adjunctive selection criterion required an overall practical parkour experience of 1.5–5 years for the beginners and at least five years for the advanced.

The study was performed following the Declaration of Helsinki and ethical national and international guidelines and regulations; each participant filled in an informed consent form, and the ethical committee of the Politecnico di Milano approved the study before experimental procedures began (opinion n. 3/2017).

### Design and Procedures

**Figure 1:**
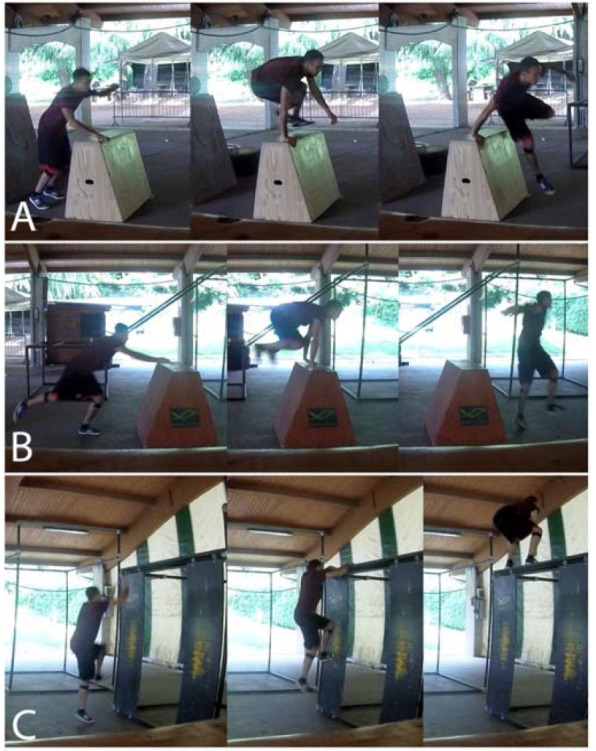
The Parkour routine adopted in this study was composed of a step vault (A), a monkey vault (B), and a wall run (C). *The traceur in the photos is one of the Authors (L.B.) of the present study, an avid parkour participant*.

Data were acquired in a sports centre parkour area, where a specific parkour path was assembled based on two parkour instructors' suggestions. We installed two plinths and a three-meter-high vertical wall to enable the execution of a sports routine composed of three typical parkour movements (MOVs), namely: a step vault (MOV1), a monkey vault (MOV2), and a wall run (MOV3) (Dvořák et al., 2018) (Figure 1).

The wall run has the specific characteristic as it requires the same pattern of ground locomotion, but applied along a vertical spatial direction (Croft et al., 2019). Before the measurement sessions, participants were invited to have a 10-min warm-up session; however, they did not have access to the path before the tests began to reduce potential learning effects. The warm-up included two minutes of in-place running, 30 s of knee raises and calf raises, and activation exercises (e.g., small jumps).

Stretches with eccentric and concentric loading were explicitly avoided since they can affect the muscles' stretch shorting cycle, resulting in post-activation potentiation during the explosive movements, thus potentially leading to biases between participants of different levels (Grosprêtre et al., 2016). Each participant underwent three consecutive trials (Trial 1, Trial 2, and Trial 3). Each trial consisted of executing the sequence of the predisposed three motor tasks (i.e., a step vault, a monkey vault, and a wall run) consecutively performed as a single course. The MIMU was started before and stopped after each trial. The purpose of the trials' repetition was twofold: concerning the study's primary goal, the aim was to enforce the inter-individual comparison between advanced participants and beginners; regarding the secondary goal, the purpose was to highlight the movement's subtle individualities or 'signature' patterns in the intra-individual analysis.

### Measures

During the trials, each athlete was wearing a MIMU placed on the back of the pelvis, through a specially designed elastic band, according to the previous experience by Seifert et al. (2014). A physician applied the MIMU to participants in correspondence with the posterior superior iliac spines (PSIS) line, which was detected through palpation. This level generally corresponds to the second sacrum vertebra, which can approximate the human body's centre of mass (CoM) during the gait (Yang et al., 2014).

We adopted the Mtw Awinda Development Kit (XSens, Enschede, The Netherlands). It includes one 3D-MIMU and a software development kit (SDK).

The MIMU weighed 16 g and measured 47 mm x 30 mm x 13 mm. The SDK provided access to 3D orientation, 3D acceleration, and 3D magnetic field data of the MIMU. The output was transmitted wirelessly using the Awinda patented communication protocol to the Awinda dongle (a USB receiver) (Paulich et al., 2018). During each session, a video of the performance was recorded by two different cameras (GoPro, Hero3+) placed orthogonally; one on the left side and one at the rear of the path.

The MIMU sampling rate was 100 Hz, while the video cameras, of which purpose was to serve as a reference tool for navigating the MIMU's data, had sample rates of 30 and 60 Hz; frequencies commonly used to assess physical activity (Migueles et al., 2017). Since the cameras and sensors had different sample rates and were manually activated, a small jump on the spot was added at the beginning of the routine for synchronization purposes. Fluency was assessed based on the existing literature; higher smoothness and smaller hesitation values (Kerr et al., 2013).

### Statistical Analysis

*Smoothness* was defined as the number of inflexions in the jerk's graph, where the jerk was the third derivative of the displacement relative to time (Hogan, 1984). Acceleration (*a*_tot_) was preliminarily calculated, for each sample of the raw signal, as follows:


$$atot=\sqrt{ax^{2}+ay^{2}+az^{2}}$$

The jerk curve was obtained from *a*_tot_, by directly calculating the jerk through derivation (jerk = d *a*_tot_/dt), using Matlab function ‘diff’. The sensor’s sample rate (f = 100 Hz) was used to determine the time (as a vector) so that it was possible to represent the jerk signal in respect of time instead of the sample number. We finally presented the jerk values on a graph through the MATLAB command ‘plot()’.

*Inflexion* refers to a change in direction over two percentage points in the CoM jerk signal, namely local maxima and minima (Hogan, 1984). We separately considered both overall inflexions and inflexions through the zero line. To calculate the total number of jerk inflexions, we adopted a MATLAB function to identify overall local maxima and local minima points.

A second MATLAB function was used to calculate through zero-line jerk inflexions. For this purpose, we accounted for the local maxima and local minima points under the condition that each maximum point must have had a positive value and been followed and preceded by a minimum of the opposed (i.e., negative) sign. On the contrary, each maximum point must have had a negative value followed and preceded by a maximum opposed (i.e., positive) sign.

*Hesitation* was defined as the CoM velocity drop during specific movement stages (Kerr et al., 2013). Indeed velocity drops are not necessarily related to hesitation, and some are expected parts of the specific movements; for example, some velocity drops are always generated when participants land after getting past obstacles. By definition, it was therefore critical to identify the specific parts of the movement relevant to the hesitation. Therefore, we started our investigation on hesitation by a comparative analysis of the videos and signal samples. For this purpose, we exploited the MATLAB video-viewer, using the initial jump on the spot to synchronize the signal samples with video frames. We identified and selected three *relevant parts* (of duration ranging between 0.8 s and 4.2 s) of the parkour routine, one for each of the three MOVs, to conduct our analysis. This process was carried out visually on the videos because we did not find any signal characteristic or automatic procedure to distinguish the corresponding frames and samples. The criteria adopted for identifying the selected parts are described in Table 1.

**Table 1:** Selected parts of each movement to study hesitation. Athletes took different steps between movements due to their different levels of expertise. Therefore, in MOV2 and MOV3, the last step before jumping was considered the beginning of the selected part.

Movements	Video		Velocity Signal	
	Start	End	Start	End
**MOV1**	Frame where athletes start bending.	Frame before athletes begin to rise, in which knees are at the maximum degree of bending.	Relative maximum before the sudden drop due to knee bending.	Absolute minimum in the same deflection.
**MOV2**	Last step before athletes jump over the second plinth.	Last frame before the first foot leaves the ground.	Relative maximum coincident with the athletes' last step before the jump over the second plinth.	Subsequent absolute minimum of deflection.
**MOV3**	Last step before jumping.	Last frame that precedes the athletes' jump.	Relative maximum coinciding with the athletes' last step.	Subsequent absolute minimum of deflection.

A specific MATLAB function was used to navigate the signals coupled with the videos. As input, the function used an eight-element array containing:

- the signal sample value associated with the first jump peak,
- the video frame of the jump peak,
- the six video frames marking the beginning and the end of the three MOVs.

Hesitation was calculated as the absolute value of the CoM velocity difference between each selected part's beginning and end. The CoM velocity was calculated by integrating the acceleration vector with the cumulative trapezoidal numerical method. A low-pass second-order Butterworth filter (cut-off frequency: 0.625 Hz) was used to correct a drift generated by low-frequency signal components. Subsequently, we calculated the absolute value of the velocity drop.

Additionally, a qualitative analysis of hesitation was carried out. Time normalization for 100 points was applied to compare and average the velocity in the selected part of each MOV. Finally, we plotted the graph of the obtained mean velocity with related error bars and standard deviations.

Finally, each movement's execution times and the number of touches on the ground between movements were manually noted watching each video.

Significance analysis of the results was carried out using MATLAB functions. Nonparametric statistics (Kolmogorov-Smirnov and Lilliefors tests) were adopted since the datasets were non-normally distributed. The Kruskal-Wallis test was run to evaluate differences between beginners and advanced athletes across different MOVs. In contrast, the Friedman test was used to assess the differences between successive repetitions of the same movement (across Trial 1, Trial 2, and Trial 3). A significance level of 0.05 was adopted. We applied the same analysis to the execution duration and the number of touches on the ground between movements.

We also adopted Cohen's *d* as a standardised effect size for measuring the difference between advanced and beginner groups' means in each movement over the different tests. Cohen’s *d* (absolute value) was calculated using Excel, and the effect size was considered appropriate with Cohen’s *d* value higher than 0.7.

Finally, the Intraclass Correlation Coefficient (ICC) was calculated for each movement in each athlete's group. For the calculation of the ICC, we used Python's Pingouin library 0.5.3; we operated the following inputs: targets: athletes numbered from 1–8 beginners; 1–9 advanced; raters: Trials (1–3); ratings (value of inflexion/hesitation) for each movement in each trial.

## Results

All the 17 enrolled athletes completed the parkour routine without interference from the measurement system. The measuring equipment was easily transported to the field, and the set-up required about fifteen minutes. When comparing advanced athletes and beginners, we found a statistically significant difference in the number of touches to the ground, comparing MOV1 and MOV2 (*p* = 6.17, 10–5) and between MOV2 and MOV3 (*p* = 4.14, 10–5). Also, the execution time was significantly different between the two groups for MOV2 (*p* = 0.002) and MOV3 (*p* = 1.17, 10–6).

The data relative to the analysis of smoothness are reported in Table 2.

**Table 2:** Results relative to smoothness.

Athlete group	Trial	Inflexions through zero line: median (Q1; Q3)			Overall inflexions: median (Q1; Q3)		
		MOV 1	MOV 2	MOV 3	MOV 1	MOV 2	MOV 3
Advanced	1	22 (20; 25.5)	40.5 (32.5; 47.5)	89 (69; 103)	41 (37; 42.5)	62.5 (55.5; 67.5)	122 (103; 166.5)
	2	22 (16.5; 25)	39 (33.5; 44.5)	93.5 (72.5; 111)	36 (30; 38)	65.5 (62; 67.5)	141.5 (97.5; 180)
	3	24 (21; 26.5)	45 (37.5; 49)	86 (57.5; 102)	38.5 (37; 41)	63.5 (59; 66)	124.5 (84.5;156.5)
Beginners	1	27 (23; 33.2)	47 (42.5; 49.5)	126 (105.7; 153.2)	44 (37.5; 51.7)	72 (58.5; 79)	190 (163.7; 219.5)
	2	28 (22.2; 33.5)	50 (32.7; 55)	115 (106; 133.5)	44 (36.5; 49.2)	70 (46.7; 75.2)	167 (146.5; 193.7)
	3	29 (22.7; 33.7)	45 (37.7; 53.5)	124 (96.5; 132.7)	43 (38.5; 49.0)	61 (50.2; 82.2)	152 (138.7; 214.2)
**Statistical analysis**		**Inflexions through zero line: median (Q1; Q3)**			**Overall inflexions: median (Q1; Q3)**		
		**MOV 1**	**MOV 2**	**MOV 3**	**MOV 1**	**MOV 2**	**MOV 3**
Differences between beginners and advanced athletes (*p*-value; Kruskal Wallis test)	**0.05**	0.22	**0.02**	0.08	0.50	**0.02**
Differences between (*p*-value; Friedman test)	Advanced	0.79	0.07	**0.04**	**0.03**	0.36	0.07
	Beginners	0.97	0.71	0.46	0.91	0.09	0.09

**Figure 2:**
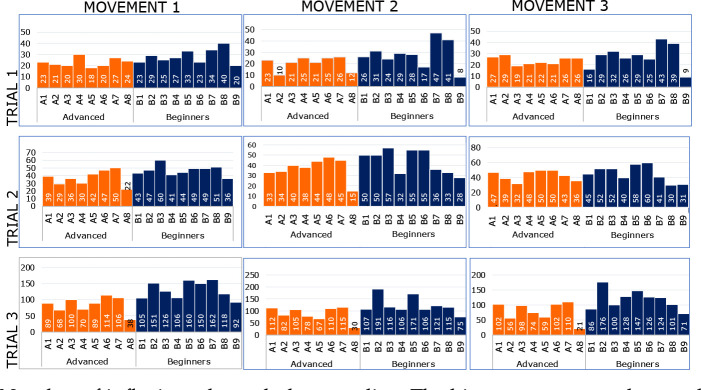
Number of inflexions through the zero–line. The histogram reports the number of overall inflexions in the jerk signal relative to the three repetitions of MOV1, MOV2, and MOV3. *The two groups are color-coded: orange for advanced; blue for beginners*.

The overall number of inflexions on the jerk graph was lower in advanced athletes than in beginners (Figure 2).

However, the number of inflexions' differences was statistically significant only relative to MOV3, both for inflexions through the zero line (*p* = 0.02) and overall inflexions (*p* = 0.02).

The mean values of the velocity drop and the relative statistical analysis are summarised in Table 3.

**Table 3:** Results relative to hesitation.

Athlete group	Trial	Hesitation (m/s) : Median (Q1; Q3)		
		Mov 1	Mov 2	Mov 3
Advanced	1	0.93 (0.82;1.61)	0.21 (0.34;0.11)	0.11 (0.25;0.07)
	2	1.17 (1.38;0.95)	0.21 (0.46;0.07)	0.20 (0.45;0.11)
	3	1.21 (1.57;1.02)	0.33 (0.51;0.17)	0.23 (0.40;0.14)
Beginners	1	1.05 (1.44;0.92)	1.43 (2.43;1.00)	0.20 (0.63;0.16)
	2	1.02 (1.09;0.93)	1.50 (2.19;0.47)	0.25 (0.70;0.15)
	3	0.95 (1.17;0.80)	0.67 (2.24;0.29)	0.28 (0.74;0.13)
Differences between beginners and advanced athletes across different MOVs (*p*-value; Kruskal Wallis Test)		0.37	4.48E-04	0.24
Differences across the three trials in the same subject (*p*-value; Friedman Test)	Advanced	0.68	0.41	0.41
	Beginners	0.45	0.45	0.12

The quantitative analysis of hesitation revealed a statistical significance only concerning MOV2 (*p* = 0.01). The Friedman tests showed no significant differences among the three subsequent repetitions of the path performed by either beginners or advanced athletes; only a few scattered values were statistically significant. The values of Cohen’s *d* are reported in Table 4, while the ICC values are reported in Table 5.

**Table 4:** The absolute value of Cohen’s *d* measuring the difference between advanced and beginner groups’ means relative to each movement in the different tests.

Inflexions through zero line		Cohen’s *d*
Mov1	Test1	0.98
	Test2	0.79
	Test3	0.46
Mov2	Test1	0.9
	Test2	0.62
	Test3	0.25
Mov3	Test1	1.79
	Test2	1.09
	Test3	1.26
**Overall inflexions**		**Cohen’s *d***
Mov1	Test1	0.79
	Test2	1.22
	Test3	0.67
Mov2	Test1	0.65
	Test2	0.01
	Test3	0.17
Mov3	Test1	1.5
	Test2	0.9
	Test3	1.07
**Hesitation**		**Cohen’s *d***
Mov1	Test1	0.07
	Test2	1.11
	Test3	0.62
Mov2	Test1	1.53
	Test2	1.07
	Test3	0.84
Mov3	Test1	0.69
	Test2	0.43
	Test3	0.58

**Table 5:** ICC values calculated for each movement in each target group.

Inflexions through the zero line				
**Advanced athletes**	ICC*	**df2**	***p***	**CI 95%**
MOV1	−0.04	14	0.550441	[–0.33, 0.5]
MOV2	0.69	14	0.000612	[0.3, 0.92]
MOV3	0.91	14	1.678326e-07	[0.73, 0.98]
**Beginners**	ICC*	**df2**	***p***	**CI 95%**
MOV1	0.79	16	0.000018	[0.49, 0.94]
MOV2	0.6	16	0.001788	[0.21, 0.88]
MOV3	0.76	16	0.000040	[0.45, 0.93]
**Overall inflexions**				
**Advanced athletes**	ICC*	**df2**	***p***	**CI 95%**
MOV1	0.56	14	0.006193	[0.12, 0.88]
MOV2	0.9	14	4.342629e-07	[0.7, 0.98]
MOV3	0.93	14	2.096574e-08	[0.8, 0.98]
**Beginners**	**ICC**	**df2**	***p***	**CI 95%**
MOV1	0.84	16	1.481438e-06	[0.61, 0.96]
MOV2	0.83	16	0.000003	[0.57, 0.95]
MOV3	0.75	16	0.000062	[0.42, 0.93]
**Hesitation**				
**Advanced athletes**	ICC*	**df2**	***p***	**CI 95%**
MOV1	0.5	14	0.013768	[0.05, 0.85]
MOV2	0.74	14	0.000186	[0.39, 0.94]
MOV3	0.64	14	0.001672	[0.23, 0.9]
**Beginners**	**ICC**	**df2**	***p***	**CI 95%**
MOV1	0.46	16	0.015079	[0.04, 0.82]
MOV2	0.72	16	0.000151	[0.37, 0.92]
MOV3	0.9	16	6.081263e-08	[0.72, 0.97]

* ICC(3, B) in accordance with the conventions by Shrout and Fleiss (1979). Specifically, the same fixed set of raters/tests (Trial 1, Trial 2, and Trial 3) rated each target group (Advanced athletes and Beginners) with no generalization to a wider population.

**Figure 3:**
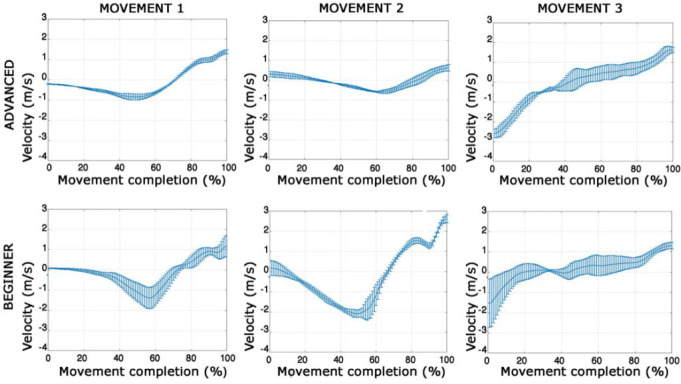
Qualitative analysis of the selected parts of the three movements (MOV1; MOV2 and MOV3) in the three trials, relative to an advanced athlete (A2) and a beginner athlete (B4).

About the qualitative analysis of hesitation, a visual examination of the error bars highlighted the movement variability at the intra-individual level over the three subsequent repetitions of the same movement during consecutive trials. In particular, while the main deflection curve shape was maintained, we found individual differences in velocity drops (Figure 3).

It was also possible to identify some general trends at an intra-individual level between athletes of the two groups. Specifically, the more advanced athletes experienced fewer velocity drops than beginners in all three movements and slower velocity reduction before the absolute minimum. Furthermore, after the absolute minimum, the curve incurred a rapid ascension, interrupted by a slight negative deflection that was generally more prominent among beginners.

## Discussion

This study aimed to explore the possibility of obtaining an objective, direct, video-assisted, single-MIMU-based measure of movement fluency in parkour, sensible enough to highlight inter-individual and intra-individual variations. Parkour is challenging to study because obstacles along a path may hinder the data acquisition through optical systems (Van der Kruk, 2018). Moreover, parkour gestures include elements of creativity and unpredictability, which, in contrast with the standard movement patterns of traditional sports (Immonen, 2017), cannot be reproduced using simulators such as, for example, the treadmill, adopted to study smoothness in running (Hreljac, 2000). Our experience prospects MIMUs as a valuable solution for acquiring data relative to movement in individual sports practised in larger space volumes and involving rapid and complex movements (Van der Kruk, 2018); parkour included.

Regarding our first hypothesis, when comparing the two groups of participants, we found a statistical difference in execution times and the number of touches on the ground between movements. Such a result confirms the existence of fundamental differences in the execution between the two groups. We also obtained a quantitative assessment of both smoothness and hesitation to compare advanced athletes and beginners.

Concerning smoothness, we found fewer overall inflexions in the jerk graph among advanced athletes than in beginners. However, the differences were significant only in the wall run, a movement which emphasizes the differences in smoothness. In the wall run, beginners included more strides in the run-up and had more movement acceleration modifications before jumping onto the top of the wall.

*Hesitation* is the delay in the movement that can appear in the motor-planning activity when choosing a novel decision task (Kaufman, 2015), and a difference between advanced and beginner participants was expected. However, the measurements only reached statistically significant values in the monkey vault (MOV2), the most complex movement. This evolution was preceded by a few preparatory steps on this path; more advanced athletes recorded lower hesitation values connecting each step before jumping over the second obstacle. On the contrary, the lack of a statistically significant difference between the two groups in MOV1 was expected. On the one hand, the step vault is a simple movement; on the other, it was the series's first movement without any need to link the action to a previous one. On the contrary, a similar result for MOV3 was surprising. A possible explanation is that the need to completely change the direction of movement, from horizontal to vertical, involves high levels of hesitation in both advanced athletes and beginners, overriding any significant difference between the two groups.

The values of Cohen's *d* confirmed that the system could reveal differences between beginners and advanced athletes in the studied parameters, particularly in MOV3 regarding smoothness and MOV2 concerning hesitation.

At the same time, the ICC results, despite one single value, fell in the fair (>0.5) to excellent (>0.9) range of intraclass correlation according to guidelines given by Koo and Li (2016), confirming that values from the same group tend to be similar.

About the secondary hypothesis, the lack of statistically significant intra-individual differences in the three trials' repetition excludes any considerable learning effect. However, in advanced athletes, such a result could be expected because, from the perspective of parkour as a discipline, the obstacle course was relatively simple, and advanced athletes would have probably performed, from the first trial, at the peak of their possibilities.

While three measurements may be insufficient to capture statistically relevant improvements even among beginners, previous studies about the effect of practice on jerk minimization have led to contradictory results (Schneider and Zernicke, 1989; Young and Marteniuk, 1997).

Furthermore, some authors reported a notable drop in the jerk values with training sessions separated by two rest days (Seifert et al., 2014). Thus, adequate rest between training sessions could probably be necessary to enhance neuromuscular control sufficiently to improve smoothness. However, it cannot be completely excluded that the system's sensitivity was insufficient to detect subtle improvements in hesitation. Finally, in the beginner group, fatigue could have negatively influenced the last measurements; however, this seems improbable, given the shortness of the adopted path.

The exclusion of any measurable learning effect in this study allows considering the subtle intra-individual differences over the three trials as an expression of intra-individual variability of movement. A substantial body of literature has highlighted how multi-articular movement variability plays a functional role in sports performance optimization in any given environment (Seifert et al., 2013). For this reason, we adopted a qualitative analysis of hesitation to highlight intra-individual (i.e., intra-trial and inter- trial) variability, to highlight those subtle movement individualities or 'signature' patterns, functional to approaching constrained motor tasks (Croft et al., 2019). The qualitative analysis of hesitation showed lower movement variability among advanced athletes, depending on these athletes' significant experience in performing the parkour's basic motor patterns. We also highlighted some recurrent differences between beginners and more advanced athletes in the monkey vault (MOV2) and the wall run (MOV3): two relatively complicated movements combined with the need for linkage to prior evolutions. Specifically, beginner athletes tended to experience more prominent velocity drops and deeper negative deflections. A possible explanation is that more skilled athletes can vary the joint biomechanical degrees of freedom in the chain of movements as the current task constraints demand. At the same time, beginners do not have the same ability and present weak adaptation to movement tasks and more non-functional variability (Davids et al., 2003; Newell and Vaillancourt, 2001).

The inclusion of a broader number of sports participants may improve statistical significance in further studies. Furthermore, the reliability of the measurement system should be confirmed in future research with a more standardized examination protocol involving sports paths requiring a more limited degree of freedom, for example, the repetition of more simple and identical movement patterns, such as jumps or steps involving the athlete approaching an obstacle with the same leg. A similar sports path could permit comparing the data of the jerk and hesitation with those provided by an optoelectronic system in a laboratory.

## LIMITATIONS

This study should be interpreted considering some limitations. First, the MIMU was placed in correspondence with the PSIS line, which generally corresponds to the CoM of the human body if the subject is in an upright position. Since the CoM moves along a 3D trajectory during whichever kind of locomotion, this can only be an approximation when studying 3D movements within a wide area (Minetti et al., 2011). This erroneousness also affects velocity and acceleration. However, since our purpose was to obtain a simple intra-individual and inter-individual comparison of fluency and movement variability rather than a precise absolute measurement of these parameters, adopting this approximation may still result in an acceptable compromise. Second, the limited number of participants and the restricted number of performed trials may also have influenced the statistical significance of the results. Another methodological limitation was the use of different sampling frequencies between the MIMU sampling at 100 Hz and the video cameras having sample rates of 30 and 60 Hz. This difference, however, did not affect the quality of the analysed signal. Indeed, the signal used for fluency analysis was that recorded by the MIMU at a sample rate of 100 Hz, while the videos obtained at a lower sample frequency only served as a reference tool for navigating the MIMU's data.

## CONCLUSIONS

This study shows that video+MIMU technology may provide a simple, direct estimation of the fluency of movement in the parkour's non-cyclical and elaborate gestures. In particular, the number of jerk inflexions through zero lines seems a more promising approach to quantitatively assess the fluency of movement.

The objective measurement of the fluency of movement may highlight the more critical phases of a sports task to target training or re-training better. Fluency measurement may guide the development of tools to enhance athletes' relations with the performance environment, for example, task simplification and decomposition or specific practice simulations (Seifert et al., 2013). In addition, after an injury, fluency assessment may assist the decision to return to sport, which is a particularly critical aspect, especially in acrobatic and high-risk sports like parkour.

About the second goal of this study, the adoption of technological advances to highlight subtle movement individualities may permit the manipulation of constraints, leading to the development of individualized movement responses focused on the intrinsic dynamics of any parkour athlete (Davids et al., 2003).

This experience was focused on parkour, but fluency assessment can be helpful in many sports disciplines. Indeed, the functional role of fluency and intra-individual and inter-individual multi-articular movement variability has been highlighted in many sports such as climbing, swimming, long and triple jumping, and in many athletic movements (e.g., soccer kicking, basketball shooting, table tennis forehand drive, volleyball serve, handball shot and field hockey drive) (Piana et al., 2016; Seifert et al., 2013). As a result, identifying slight differences in fluency between athletes and characterizing individual fluency variations between athletic gestures and subsequent repetitions can allow adapting the intrinsic dynamics of particular learners by enhancing their relations with the performance environment.

Practical future development might involve designing a system with which trainers and coaches could visually evaluate athletes' performance and immediately identify fluency differences.
